# Etiologic evaluation and pregnancy outcomes of fetal growth restriction (FGR) associated with structural malformations

**DOI:** 10.1038/s41598-024-59422-8

**Published:** 2024-04-22

**Authors:** Xiaoqing Wu, Shuqiong He, Qingmei Shen, Shiyi Xu, Danhua Guo, Bin Liang, Xinrui Wang, Hua Cao, Hailong Huang, Liangpu Xu

**Affiliations:** 1 Fujian Maternity and Child Health Hospital College of Clinical Medicine for Obstetrics & Gynecology and Pediatrics, Fujian Medical University, No.18 Daoshan Road, Fuzhou City, 350001 Fujian Province China; 2 Fujian Provincial Key Laboratory for Prenatal Diagnosis and Birth Defect, Fuzhou, China; 3 Department of Laboratory Medicine, Fujian Medical University, Fuzhou, China; 4 Key Laboratory of Clinical Laboratory Technology for Precision Medicine (Fujian Medical University), Fuzhou, China; 5 Fujian Provincial Hospital, Fuzhou, China

**Keywords:** Fetal growth restriction, Structural malformations, Karyotyping, Single nucleotide polymorphism, Cytomegalovirus, Copy number variants, Cytogenetics, Genetic association study, Genomics, Diagnosis

## Abstract

This study aimed to evaluate the etiology and pregnancy outcomes of fetuses underwent invasive prenatal diagnosis for fetal growth restriction (FGR) accompanied by structural malformations. Data from 130 pregnancies referred for prenatal diagnosis for FGR accompanied by structural malformations were obtained between July 2011 and July 2023. Traditional karyotyping was conducted for all the subjects. A total of 37 (28.5%) cases of chromosomal abnormalities were detected by karyotyping, including 30 cases of numerical anomalies and seven cases of unbalanced structural anomalies. Trisomy 18 was the most common abnormalities, accounting for 51.4%, significantly higher than any other chromosomal abnormality. The cohort was predominantly comprised of early-onset FGR (88.5%) compared to late-onset FGR (11.5%). The incidences of chromosomal abnormalities in this two groups were 29.6% (34/115) and 20.0% (3/15), respectively (*p* > 0.05). The majority (74.6%, 97/130) of the cohort were affected by a single system malformation, with chromosomal abnormalities found in 19.6% (19/97) of cases. In pregnancies of structural malformations involving two and multiple systems, the frequencies were 56.5% (13/23), and 50.0% (5/10), respectively. Single nucleotide polymorphism array (SNP array) was performed in parallel for 65 cases, revealing additional 7.7% cases of copy number variants (CNVs) compared to karyotyping. Polymerase chain reaction (PCR) was used for detection of cytomegalovirus (CMV) DNA in 92 cases. All fetuses with FGR associated with two or more system malformations were either terminated or stillborn, irrespective of chromosomal aberrations. Conversely, 71.8% of pregnancies with a single-system malformation and normal genetic testing results resulted in live births. Furthermore, two (2.2%) cases tested positive for CMV DNA, leading to one termination and one case of serious developmental disorder after birth. Our study suggests that structural malformations associated with FGR are more likely to affect a single organ system. When multiple systems are involved, the incidence of chromosomal abnormalities and termination rates are notably high. We advocate for the use of CMA and CMV DNA examinations in FGR cases undergo invasive prenatal diagnosis, as these tests can provide valuable insights for etiological exploration and pregnancy management guidance.

## Introduction

Fetal growth restriction (FGR) is a condition that fetus does not reach its intrauterine biological potential for growth and development. Traditionally, FGR has been defined as fetuses with an estimated fetal weight (EFW) below the 10th percentile for gestational age, and it could be symmetric or asymmetric. Some FGR symmetric may be actually normal small for gestational age (SGA), and many fetuses diagnosed with FGR exhibit have unexpectedly normal growth afterbirth^1–4^. Nonetheless, when FGR is suspected, it is suggestive to conduct an etiology evaluation to assess the prognosis and make informed decisions regarding the pregnancy.

Multiple factors have been implicated in FGR. Maternal factors and uteroplacental factors are primarily related to obstetric management, while Genetic diseases play a significant role in fetal factors, often necessitating invasive prenatal genetic diagnosis. In recent decades, convention karyotyping and chromosomal microarray analysis (CMA) have become widely accepted for the routine genetic diagnosis of FGR, revealing a range of chromosomal and submicroscopic disorders. Survival data for growth-restricted fetuses without structural defect have been well-documented^5–7^. In our previous study related to genetic findings of FGR without structural malformations, karyotyping detected chromosomal abnormalities in 3.9% of cases, while CMA identified an additional 4.2% with clinically significant submicroscopic aberrations^8^. In clinical practice, the co-occurrence of FGR and structural malformations is a common observation, yet specific estimations of etiology are seldom reported. The current study retrospectively reviews the profiles of malformations, genetic etiology, and pregnancy outcomes in 130 pregnancies affected by both FGR and structural abnormalities. Additionally, considering that congenital cytomegalovirus (CMV) infection is the most relevant infection factors for FGR^9,10^, the quantitative determination of CMV DNA in 92 cases of prenatal specimens was analyzed. The purpose of this study is to explore the etiology and pregnancy outcomes in pregnancies with FGR complicated by structural malformations. We hope to provide a more comprehensive understanding of the etiology and pregnancy outcomes in cases of FGR complicated by structural malformations. This work builds upon our previous publication on FGR and will aid in guiding clinical consultations.

## Materials and methods

### Patients and samples

The retrospective data were collected between July 2011 and July 2023, encompassing 130 pregnancies that underwent invasive prenatal diagnosis due to the diagnosis of FGR accompanied by structural malformations. Among these, 92 cases were identified as symmetric FGR, and 38 cases were classified as asymmetric FGR. FGR was diagnosed based via ultrasound when EFW fell below the 10th percentile based on the Hadlock formula. The gestational age at FGR initially diagnosed was 25.8 ± 4.5 weeks, with 88.5% of cases being diagnosed before 32 weeks (early-onset FGR) and 11.5% diagnosed after 32 weeks (late-onset FGR). Detailed anatomical scans were performed when FGR was diagnosed. Structural abnormalities affected various organ systems, including the cardiac, craniocerebral, gastrointestinal, genitourinary, skeletal, and faciocervical systems. Depending on the number of organ systems involved in the structural malformation, they were further classified into groups of single-system, two-system, and multiple-system malformations. The basic characteristics are presented in Table 1.

**Table 1:** Basic characteristics of the 130 FGR pregnancies.

Characteristic	Value
Gestational age at invasive prenatal diagnosis (weeks), mean (SD)	23.8 (2.8)
Gestational age at FGR onset	
< 32, n (%)	115 (88.5%)
≥ 32, n (%)	15 (11.5%)
Maternal age (years), mean (SD)	28.0 (5.1)
≥ 35, n (%)	14 (10.8%)
< 35, n (%)	116 (89.2%)
Structural Malformations	
Single system, n (%)	95 (73.1%)
Cardiac, n	59
Craniocerebral, n	13
Genitourinary, n	9
Gastrointestinal, n	6
Skeletal, n	4
Faciocervical, n	4
Two system, n (%)	23 (17.7%)
Multiple system, n (%)	12 (9.3%)

Our samples included 70 cases of amniotic fluid and 60 cases of umbilical cord blood. Among them, 25 cases of umbilical cord blood was collected during induction of labor.

### Methods

All experiments were performed in accordance with relevant guidelines and regulations.

### Cytogenetic analysis

Karyotyping was performed on all the subjects. The cytogenetic analysis progress involving cell culture and G-banded karyotyping was performed according to the standard protocols in local laboratory, similar to those described in previous publication^11^. The karyotype was analyzed at a resolution of 320–500 bands level, using International System international system for human cytogenetic nomenclature 2020 (ISCN 2020)^12^ for karyotype description. Numerical chromosomal abnormalities and unbalanced structural abnormalities by conventional karyotyping were deemed clinically significant.

### SNP array analysis

SNP array analysis has been utilized in our center since late 2016, thus only 65 pregnancies underwent SNP array analysis in parallel. Genomic DNA was extracted from uncultured amniotic fluid and cord blood samples using QIAGEN kit (Qiagen, Germany), following the manufacturer’s instructions. We employed the Affymetrix CytoScan 750 K array (Affymetrix Inc., Santa Clara, CA, USA) for SNP array analysis. This array includes 200,000 probes targeting single nucleotide polymorphisms and 550,000 probes designed to detect copy number variations (CNVs) throughout the entire human genome. As described in our prior publication^8^, microarray-based CNV analysis was performed using Chromosome Analysis Suite software (ChAS), version 3.1 (Affymetrix, Santa Clara, CA, USA), and genomic imbalances were annotated based on the GRCh37/hg19 Genome Build (July 2013). We maintained a general threshold of significance: gains or losses of ≥ 400 kb and regions of homozygosity (ROH) ≥ 10 Mb. Uniparental disomy (UPD) was identified based on the presence of a region of homozygosity (ROH) encompassing an entire chromosome. A specialized UPD tool was employed for comprehensive genome-wide detection of UPD within child-parent trios to confirm the maternal or paternal origin of UPD.

All identified CNVs were cross-referenced with both our institutional database and national public CNV repositories, including the Database of Genomic Variants (DGV), Database of Chromosome Imbalance and Phenotype in Humans Using Ensemble Resources (DECIPHER), International Standards for Cytogenomic Arrays Consortium, and Online Mendelian Inheritance in Man (OMIM).

The results from SNP array analysis was analyzed following the definition provided by the American College of Medical Genetics (ACMG)^13^, and were categorized into five levels: pathogenic, benign, likely pathogenic, likely benign, and variants of uncertain significance (VOUS). Clinically significant findings included pathogenic and likely pathogenic variants. Parental SNP array analysis was recommended to ascertain the inheritance of CNVs.

### CMV-DNA testing

CMV DNA was extracted from amniotic fluid or cord blood on the Magna Pure LC Instrument (RocheMolecular Biochemicals, Meylan, France) with the Total NA serum-plasma kit (Roche Diagnostic). The viral load was assessed through quantitative polymerase chain reaction (qPCR) analysis. A viral load of ≥ 500copies/ml was considered as a positive result.

### Pregnancy outcomes follow up

Information on pregnancy outcomes, including stillbirth, termination of pregnancy (TOP), and live birth, was collected from the hospital’s clinical database or through direct telephone inquiries. The follow-up ages ranged from 2 months to 5 years.

### Statistical analysis

The data were analyzed using SPSS software v26.0 (SPSS Inc., Chicago, IL, USA). Statistical comparisons were performed using the chi-square test, the Fisher’s exact test, and *p* < 0.05 was considered statistically significant.

### Ethical approval and consent to participate

The present study was approved by the Protection of Human Ethics Committee of Fujian Provincial Maternity and Children’s Hospital. Written informed consent was obtained from each pregnant woman.

## Results

As outlined in Table 2, among the 130 pregnancies associated with structural malformations, a total of 37 (28.5%) chromosomal abnormalities were identified, including 30 numerical aberrations and seven cases of unbalanced structural abnormalities. The most common aberration was trisomy 18, accounting for 51.4% (19/37), followed by trisomy 21 (8.1%) and trisomy 13 (8.1%). The frequencies of chromosomal abnormalities between early-onset FGR and late-onset FGR showed no significant differences (29.6% vs. 20.0%, *p* > 0.05).

**Table 2:** Details of chromosomal abnormalities by karyotyping among 130 cases.

Chromosomal abnormality	Value
Numerical abnormality, n (%)	30 (23.1)
Trisomy 18	19
Trisomy 21	3
Trisomy 13	3
Triploidy	2
45,X	1
48,XXX, +18	1
47,XYY	1
Unbalanced structural abnormality, n (%)	7 (5.4)
Total, n (%)	37 (28.5)

Table 3 displayed the types and chromosomal abnormality frequencies in pregnancies with anomalous FGR. The majority of FGR cases (73.1%) involved structural malformation in a single organ system, followed by two-system involvement (17.7%), and multiple-system involvement pregnancies (9.3%). Their rates of chromosomal abnormalities were 18.6% (18/95), 56.5% (13/23), and 50.0% (6/12), respectively. Cardiac malformations were the most common type, occurring in 93 pregnancies, with 59 of them being affected only by cardiac malformation, showing a chromosomal abnormality rate of 18.6%.

**Table 3:** Chromosomal abnormalities by karyotyping for pregnancies complicated by FGR and different structural malformations.

Organ system involved in malformation	Incidence of chromosomal abnormalities
Single system	18.6%, 18/95
Cardiac	18.6%, 11/59
Craniocerebral	23.1%, 3/13
Genitourinary	11.1%, 1/9
Gastrointestinal	16.7%, 1/6
Skeletal	25.0%, 1/4
Faciocervical	25.0%, 1/4
Two systems	56.5%, 13/23
Multiple systems	50.0%, 6/12

Among the 65 cases underwent both karyotyping and SNP array in parallel, additional 6 cases of submicroscopic aberration were revealed by SNP array compared to karyotyping, with 5 (7.7%) cases being clinically significant. Detailed information is presented in Table 4. Three of these cases were related to known syndrome: 15q24 Microdeletion Syndrome (#613,406), 8q21.11 Microdeletion Syndrome (#614,230), and DiGeorge Syndrome (#188,400/#192,430).

**Table 4:** Submicroscopic aberrations with clinical relevance by SNP array analysis.

Case number	Ultrasound findings	Gestational age at FGR first diagnosed (weeks)	Karyotyping results	CMA results	Type of aberration/size	Inheritance	Related syndrome/pathogenic classification	Pregnancy outcome
1	FGR, VSD	17	46,XY	arr[GRCh37] 3q26.33q27.2(182,374,672–185,041,523) × 1	del/2.6 Mb	De novo	Pathogenic	TOP
2	FGR, VSD, pulmonary valve stenosis with tricuspid valve insufficiency	21	46,XX	arr[GRCh37] 15q24.1q24.2(72,965,465–75,567,135) × 1	del/2.6 Mb	De novo	15q24 Microdeletion Syndrome/Pathogenic	TOP
3	FGR, aortic coarctation, increased cardiothoracic ratio, pericardial effusion, fetal NF thickening, fetal spinal curvature increased	21	46,XY,inv(11)(p15q21)dn	arr[GRCh37] 5q22.3q23.1(113,627,122–116,240,273) × 1, 8q21.11q21.13(74,350,927–81,710,386) × 1	5: del/2.6 Mb 8: del/7.3 Mb	De novo	8q21.11 Microdeletion Syndrome/Pathogenic	Still birth
4	FGR, VSD, aortic stenosis; Hypoplastic or absent left kidney	23	46,XY	arr[GRCh37] 16q23.2q24.3(79,800,878–90,146,366) hmz, 16p13.3p12.3(94,807–19,302,326) hmz [upd(16)mat]	LOH/10.3 Mb, 19.2 Mb	De novo	Pathogenic	TOP
5	FGR,VSD	31	46,XY	arr[GRCh37] 22q11.21(18,648,855–21,800,471) × 1	del/3.1 Mb	De novo	DiGeorge Syndrome or velocardiofacial syndrome /Pathogenic	TOP

FGR, fetal growth restriction; VSD, ventricular septal defects.

CMV infection was confirmed in 2 (2.1%, 2/92) cases who both exhibited craniocerebral malformation. Fetus 1 manifestied FGR, cerebral cortical dysplasia, Blake’s pouch cyst, posterior fossa abnormalities, and ventriculomegaly. The fetus resulted in live birth, and the child suffered from severe speech, hearing, and motor impairments at 4-years follow-up. Fetus 2 had FGR, severe ventriculomegaly, and broadening cisterna magna, and was finally terminated.

Pregnancy outcomes were available for 126 cases (95.4%). All pregnancies with clinically relevant genetic aberrations were terminated. The outcomes of the remaining 84 cases with normal genetic testing are shown in Table 5. All 13 cases with two and multiple system malformations ended in TOP. In the 71 pregnancies complicated by FGR and a single system malformation, 51 (71.8%) resulted in live births. Normal development was observed in 49 of them. The rest two cases showed abnormal development: one was of CMV infection, as mentioned above (fetus 1); the other one, with physical development delay, was found in case of sever FGR (EFW < 3th percentile) and duodenal stenosis.

**Table 5:** Outcomes for 84 FGR pregnancies without genetic abnormalities.

Malformations accompanied by FGR	Live birth, n (%)	Still birth, n (%)	TOP, n (%)
Single-system malformation (N = 71)	51 (71.8)	3 (4.2)	17 (23.9)
Two system malformation (N = 7)	0 (0.0)	0 (0.0)	7 (100.0)
Multiple system malformation (N = 6)	0 (0.0)	0 (0.0)	6 (100.0)
Total	51 (61.4)	3 (3.6)	29 (34.9)

## Discussion

As expected, fetuses with FGR complicated by structural malformations were at a high risk of chromosomal abnormalities. The incidence of chromosomal abnormalities by karyotyping was 28.5%, much higher than 3.9% in our previous study on FGR without structural malformation^8^. Trisomy 18 was the most frequently encountered aberration, similar to the finding in the manyreports^5,11,14^. Additionally, many studies have reported a decrease in the rate of chromosomal abnormalities in isolated FGR fetuses as the gestational age at which FGR is first diagnosed increases, with fewer or no chromosomal abnormalities observed in pregnancies with isolated FGR diagnosed after 32 gestational weeks^8,16,17^. According to the latest SMFM (Society for Maternal–Fetal Medicine) guideline, late-onset FGR is not considered an indication for invasive diagnosis^18^. However, our study demonstrated that fetuses with FGR complicated by structural malformations diagnosed before and after 32 weeks showed similarly high detection rates (29.6% and 20.0%) of chromosomal anomalies. Therefore, we suggest that genetic evaluation should be considered when structural malformations is present in late-onset FGR.

We explored the influence of malformations, and found that the frequencies of genetic defects and pregnancy outcomes depended on the number of organ system involved in malformations. In FGR with a single-system malformation, the incidence of microscopic abnormalities (18.9%) was much lower than those involving two or multiple systems malformations (56.5% and 50.0%, respectively). Among pregnancies with follow-up data available, all those with two or more systems malformations were terminated, regardless of the presence of genetic abnormalities, whereas live births were observed in 72.9% of single-system malformation pregnancies. The findings demonstrate that when there are more than two system malformations involved in FGR, the rates of chromosomal abnormalities and pregnancy termination are extremely high. The cardiac system was most frequently involved, affecting up to 62.1% of the single-system malformation group, followed by craniocerebral system (13.7%) and genitourinary system (9.5%). Its rate of chromosome abnormalities was close to those involving other systems. Many scholars are concerned about changes in the structure and function of the cardiac and craniocerebral system of FGR fetuses, as they may be related to short-term and long-term adverse effects on FGR fetuses^19,20^. In this study, we focused on the genetic etiology, and we found that the rates of chromosomal abnormalities were similar between them.

All live births were from pregnancies involving single systemic malformation. Only two out of 71 survivors exhibited abnormal phenotypes. One of them had complex craniocerebral malformation in prenatal ultrasound. The clinical features of severe speech, hearing, and motor impairments after birth can be largely explained by intrauterine CMV infection in this case. Intrauterine CMV infection often lead to craniocerebral malformation, which was present in both two cases with CMV infection in our study, and can lead to a series of serious developmental disorders after birth^9,21^. The intrauterine manifestation and postnatal phenotype of this case highlighted the significant harm of intrauterine CMV infection and the potential association of CMV infection with FGR^10,22^.

An increase in pathogenic CNVs has been recognized to associate with FGR. In our previous study on FGR without structural malformations, SNP array analysis yielded additional 4.2% of clinically relevant aberrations compared with karyotyping^8^. In current study, FGR pregnancy with structural anomalies showed a slightly higher value of 7.7%. The finding was similar to that reported by Schaeffer et al.^23^. However, in a recent study by Chen et al.^24^, the pathogenic CNVs detection rate in FGR with structural anomalies was as high as 33.33%. The significantly varied results may be explained by different sample size and different FGR definition standards. Three known syndromes were involved in our study. Among them, 15q24 Microdeletion Syndrome (#613,406) and 8q21.11 Microdeletion Syndrome (#614,230) were rarely characterized by FGR^25^. As for 22q11.21 microdeletion, which is responsible for DiGeorge Syndrome or Velocardiofacial Syndrome, FGR was frequently reported in FGR with structural malformations^12,26–28^. In one case (case 1) presenting FGR and ventricular septal defect (VSD), a 2.6 Mb deletion was detected in the region of 3q26.33-3q27.2, involving 30 OMIM genes. This aberration is a rare condition in which all previously reported cases have experienced FGR and some other phenotypes^29–32^. In addition, maternal UPD of chromosome 16 was revealed in a fetus with FGR, VSD, aortic stenosis, and hypoplastic or absent left kidney. According to existing database and literatures, UPD (16) has been well believed to be correlated with FGR, mainly due to its potential impact on the function of the placenta^33–35^. This further strengthens the practical value of CMA in the etiological diagnosis of FGR and the associated malformations.

Our study was limited by the small sample size. In addition, not all cases underwent SNP array analysis and CMV DNA testing, which may introduce bias in etiology evaluation of submicroscopic aberration and intrauterine infection. A study with a larger sample size, comprehensive examination, and long-term follow-up is required to accurately assess the etiology and prognosis of fetuses with FGR associated with structural abnormalities.

In conclusion, structural malformations associated with FGR were more likely to involve a single organ system. When more than one system is involved, the incidence of chromosomal abnormalities and pregnancy termination is very high. In cases of invasive prenatal diagnosis, we recommend conducting CMA and CMV DNA examinations for etiological exploration and guidance in pregnancy management.

## Data Availability

The datasets used and/or analyzed during the current study are available from the corresponding author on reasonable request.
